# Vulnerability and agency across treatment-seeking journeys for acutely ill children: how family members navigate complex healthcare before, during and after hospitalisation in a rural Kenyan setting

**DOI:** 10.1186/s12939-020-01252-x

**Published:** 2020-08-10

**Authors:** Scholastica M. Zakayo, Rita W. Njeru, Gladys Sanga, Mary N. Kimani, Anderson Charo, Kui Muraya, Haribondhu Sarma, Md. Fakhar Uddin, James A. Berkley, Judd L. Walson, Maureen Kelley, Vicki Marsh, Sassy Molyneux

**Affiliations:** 1 KEMRI-Wellcome Trust Research Programme, P.O Box 230, Kilifi, 80108 Kenya; 2 Nutrition and Clinical Services Division, icddr,b, Mohakhali, Dhaka, 1212 Bangladesh; 3 Centre for Tropical Medicine and Global Health, University of Oxford, Oxford, UK; 4 Department of Global Health, University of Washington, Washington, USA; 5 The Ethox Centre, Nuffield Department of Population Health, University of Oxford, Oxford, UK

**Keywords:** Vulnerability, Agency, Treatment-seeking, Childhood acute illness

## Abstract

**Background:**

Child mortality rates during hospitalisation for acute illness and after discharge are unacceptably high in many under-resourced settings. Childhood vulnerability to recurrent illness, and death, is linked to their families’ situations and ability to make choices and act (their agency). We examined vulnerability and agency across treatment-seeking journeys for acutely ill children and considered the implications for policy and practice.

**Method:**

A qualitative sub-study was embedded within the prospective CHAIN Network cohort study, which is investigating mechanisms of inpatient and post-hospital discharge mortality among acutely ill young children across a spectrum of nutritional status. Primary data were collected from household members of 20 purposively selected cohort children over 18 months through formal interviews (total *n* = 74), complemented by informal discussions and observations. Data were analysed using narrative and thematic approaches.

**Results:**

Treatment-seeking pathways were often long and complex, particularly for children diagnosed as severely malnourished. Family members’ stories reveal that children’s carers, usually mothers, navigate diverse challenges related to intersecting vulnerabilities at individual, household and facility levels. Specific challenges include the costs of treatment-seeking, confusing and conflicting messaging on appropriate care and nutrition, and poor continuity of care. Strong power inequities were observed between family members and health staff, with many mothers feeling blamed for their child’s condition. Caregivers’ agency, as demonstrated in decision-making and actions, often drew on the social support of others but was significantly constrained by their situation and broader structural drivers.

**Conclusion:**

To support children’s care and recovery, health systems must be more responsive to the needs of families facing multiple and interacting vulnerabilities. Reducing incurred treatment costs, improving interpersonal quality of care, and strengthening continuity of care across facilities is essential. Promising interventions need to be co-designed with community representatives and health providers and carefully tested for unintended negative consequences and potential for sustainable scale-up.

## Background

Despite an overall reduction of child mortality rates in low and middle-income countries (LMICs), mortality in vulnerable subgroups remains unacceptably high [1]. While most children who present to hospital with severe illness survive the acute episode, mortality after discharge from the hospital often equals or even exceeds inpatient mortality [2, 3]. Undernourished young children with acute illness have a particularly high risk of death during hospital admission and after discharge [2, 4]. There is a clear need for a better understanding of mechanisms underlying severe illness and mortality in young children, including the clinical, nutritional, health system, environmental, socio-cultural and economic dimensions [1, 5].

To better understand treatment-seeking journeys of acutely ill children, and identify potential points of intervention, a demand-side approach where we learn from the perspectives, priorities and experiences of individuals, households and communities can be instructive [6]. Whilst some studies have examined children’s journeys into hospital and post-discharge, we are not aware of any publications longitudinally documenting family members’ perspectives before, during and after admission to hospital in LMICs. The available literature suggests that children’s pathways through care, particularly for chronic problems, including undernutrition, can be complex and dynamic [7, 8]. Important influences at household and community levels include the perceived cause and severity of a child’s symptoms and the health, wealth and well-being of the child’s main carer(s). Social relationships within and beyond the home, and availability and access to support from family and community-based organisations can also play an important role in treatment-seeking decisions, including in providing advice, accessing necessary funds, or supporting parents with childcare and other tasks [9]. At the health service or system level, influences on treatment-seeking pathways include how far away different types of services are located, their costs, and the perceived quality for the symptoms observed [10–12]. All of these influences are in turn shaped by structural drivers such as access to employment and availability of essential services.

Treatment-seeking pathways and factors influencing these journeys can reflect and reveal different types and levels of vulnerability and agency. Definitions of vulnerability and agency in the ethics and social science literature are complex and contested [13–15]. Rogers and colleagues [16] offer a useful framework and typology in feminist bioethics to carefully distinguish more than ordinary vulnerability in situations like this. They distinguish between vulnerability that is *inherent*, or intrinsic to the human condition, that arises from “our corporeality, our neediness, our dependence on others, and our affective and social natures”, and vulnerability that is more context-specific and *situational*; with the latter caused or exacerbated by the temporary or enduring conditions in which people find themselves [17]. People’s ‘agency’, or their ability to make choices and to act, has often been seen in contrast to vulnerability. However, recent research has shown how expressions of agency can arise through experiences of vulnerability, and vice versa, so that individuals can experience manifestations of both simultaneously [15]. For example, Mizen and Ofosu-Kusi [18] show that the cause of one Ghanaian girl’s vulnerability, a sexually abusive father, led her to develop strategies such as feigned illness to avoid having to go to see him. These strategies demonstrate a form of agency, albeit from a position of weakness, which potentially expose her to new vulnerabilities. Vulnerability and agency should, therefore, be seen as multiplicitous and mutually constituted, arising from the intersection and interrelatedness of ‘cultural, social, historical, political, institutional and material’ contexts [19]. These contexts can promote and constrain the nature and level of individuals’ agency. In the context of child illness, treatment-seeking pathways can be an outcome of the interplay between different forms of vulnerability and agency.

Treatment-seeking literature from Kenya and other LMICs suggests that children’s susceptibility to recurrent serious illness, disability and death is likely to be intricately linked to the vulnerability of the family members who care for them, as well as family members’ ability to make choices and to act even in constrained contexts [7, 9, 12, 20–23]. A better understanding of the specific types of vulnerability and agency experienced across treatment-seeking pathways for acutely ill children before and after hospitalisation, particularly from the direct perspective of family members, has the potential to reveal vulnerabilities that could be reduced through future practice and policy, and agency that might be built upon.

In this paper, we share family members’ perspectives on how they navigated their young children’s treatment-seeking journeys into a public hospital, through care, and after discharge from hospital in a primarily rural Kenyan setting. We examine the different forms of vulnerability and agency revealed through the treatment-seeking patterns followed for acutely ill hospitalised children and consider the implications for policy and practice.

## Methods

### The Childhood Acute illness and Nutrition (CHAIN) network

CHAIN (www.chainnetwork.org) is a multi-disciplinary research network aimed at understanding the mechanisms contributing to high mortality in hospital and after discharge in LMICs in order to identify interventions to improve survival [5]. The Network is leading a cohort study at nine hospital sites in Africa and South Asia, recruiting more than 3000 acutely-ill children at admission to hospital and following them up post-discharge. Scheduled follow up visits are conducted at days 45, 90 and 180 after hospital. Children are enrolled and classified in three strata by anthropometric status since this is a strong marker of survival risk that encompasses both biological and social risks: severe wasting or kwashiorkor (oedematous malnutrition) (SWK), moderate wasting (MW) and no wasting (NW). Enrolled children faced varied social risks in terms of social disruption, household locations and types, and levels of maternal education [5]. Audit and training were provided to sites to ensure treatment and referral for outpatient nutritional and other care after discharge was according to current national and WHO guidelines. The primary outcome of the cohort study is mortality.

### The qualitative sub-study in CHAIN

Kilifi, Kenya is one of four CHAIN sites in which in-depth qualitative work was conducted to better understand family, community and stakeholders’ perspectives on children’s illness trajectories. This qualitative research was conducted in collaboration with Resilience, Empowerment and Advocacy for Women’s and Children’s Health Research (REACH), a collaborative research ethics project aimed at better understanding the practical ethical challenges of research involving populations considered to be vulnerable. Pooled data from all four sites will be reported separately. Here we consider, in-depth, the findings from Kilifi, one of Kenya’s poorest counties. The county’s health system and population face significant structural vulnerabilities, including 68% of the population living below the poverty line [24], a shortage of health workers, regular drug stock-outs and health facility access challenges [25, 26]. Most of the population depend on small scale farming, and high levels of gender inequity have also been documented [23, 27]. The latter power relations have been shown to have important implications for children’s treatment-seeking [9].

Qualitative work in Kilifi included interviews with family members of 20 purposively selected children from the site cohort of approximately 500. Children were selected to maximise the diversity of experience, based on nutritional status, household socio-economic status, geographical location, and any recent exposure to a socially disruptive event such as death of a caregiver. Members of families who consented were visited in their homes at least three times over an 18-month post-discharge period, with most visits lasting one to three hours. A total of 74 interviews were held with the children’s primary caregivers and other family members (mostly mothers, fathers, mothers-in-law, aunts, uncles and fathers in law) between April 2017 and July 2018. Most household visits and interviews were conducted by two social science team members, with formal interviews organised to fit around domestic activities to minimise disruption to family life.

**Fig. 1:**
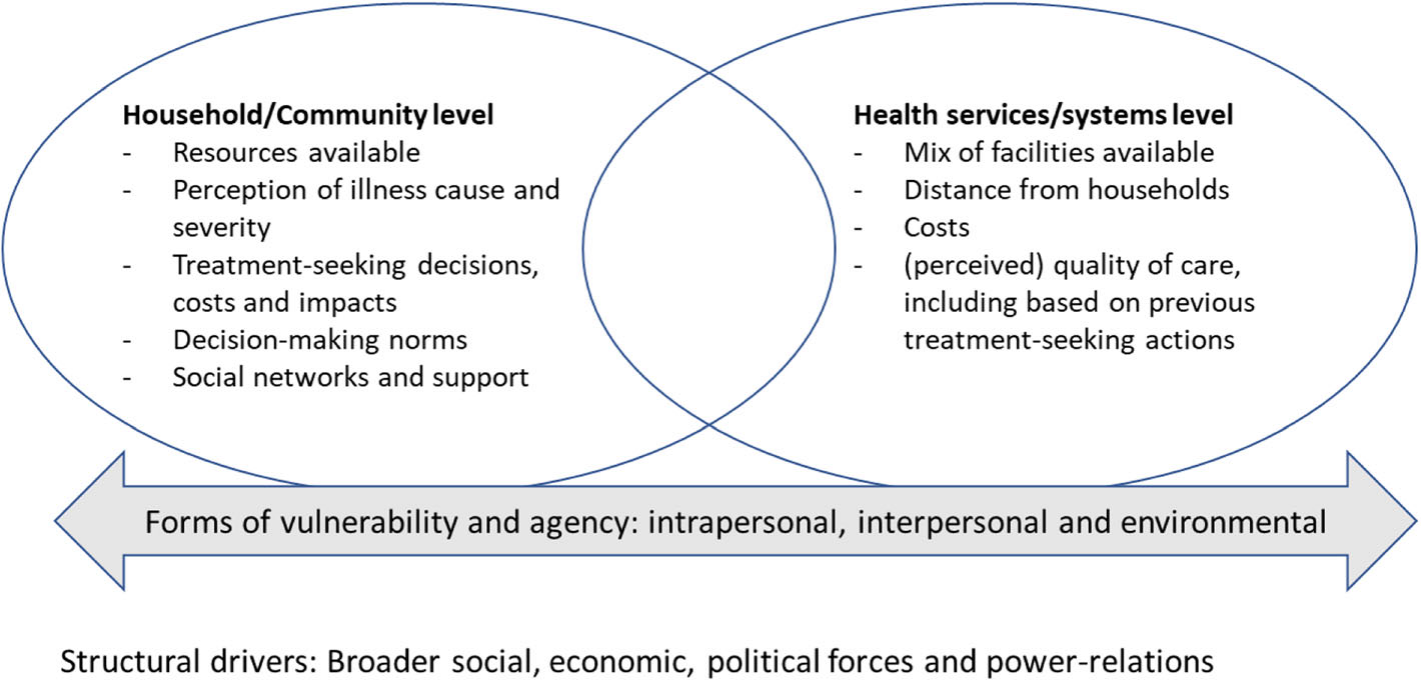
Potential influences on treatment-seeking and outcomes

Interviews covered a broad range of topics informed by the theoretical and empirical literature summarised in Fig. 1, which served as a conceptual framework. This conceptual framework incorporates ideas from the treatment-seeking literature [9, 12, 20] with Rogers et al. [16] and Lange et al.’s [17] vulnerability frameworks. For vulnerabilities, we were particularly interested in situational, more immediate, *intrapersonal* (biological or psychological), *inter-personal* (roles, relationships and interactions), and *environmental* (socio-economic and cultural, and institutional) vulnerabilities. We recognised that these vulnerabilities, and agency, across household/community and health service levels, would be shaped by broader social, economic and political forces and power-relations, or structural drivers. Quantitative data on measurable elements of vulnerability across all CHAIN sites will be presented in a future publication.

Interview guides were used flexibly to support a rich and relaxed discussion in either Kiswahili or the local dialect, Kigiriama by native speakers conducting interviews. To support data quality, interview guides were translated, piloted and revised by the research team prior to data collection. Discussions focused on experiences and decision making at each stage of the illness, any challenges encountered, coping strategies, and how these strategies were perceived to work. The overall aim was to build the story of the illness and associated treatment-seeking from the perspective of parents and other caregivers and to understand the types of vulnerabilities to (re) hospitalisation, recurring illness or death, and agency revealed through these narratives.

### Data management and analysis

All formal interviews were audio-recorded, transcribed verbatim and later translated into English. All notes from the field and post-interview debrief were typed up and reviewed for issues to follow up in later interviews.

We worked as a team to adopt two complementary approaches to analyse the data: a narrative approach [28, 29] and a thematic coding approach [30]. The narrative approach involved the construction of a detailed overall summary for each household, drawing on all available data, and a shorter narrative, or story. We worked with these summaries and stories to explore the overall picture of households’ pathways through care, examining household/community and health service/system influences pre- and post-admission. We investigated changes over time, and patterns of similarity and difference across households, facilitated by the construction of charts (see Table 1 for an excerpt). The thematic coding supplemented and enriched the narrative analysis. All transcriptions were coded in NVivo 10 using a coding framework based on our initial and emerging themes of interest, including treatment-seeking patterns and influences on those patterns. To support the trustworthiness of the coding process, at least two people coded each transcript, comparing results and resolving any discrepancies.

**Table 1:** Example of Household Charts comparing themes

PID	Length of illness	Length of treatment-seeking	Patterns of treatment-seeking	Influences on treatment-seeking				Other influences	Infor/advice on discharge & adherence
				Nature of illness and perceptions of it	Levels of access to cash	Social support	Health systems issues/referrals		
Hh3 Male, with SWK, 21 months old & unknown birth weight.	Approx. 3 months pre, 2 weeks admission, 4 months post.	About 8 months: Child occasionally gets convulsions, but the cause not clearly understood.	**PRE** Health centre-Health centre-Private clinic-Private clinic-(both retirees living in the village) -Public dispensary-duka **POST** Supp Public Dispensary-Private clinic-illness continues (child still not well), duka, Public dispensary, Private clinic.	Believed uvula was causing vomiting, diarrhoea and loss of appetite. Symptoms persisted after it was traditionally cut. Afterwards, diviner diagnosed possession by some evil spirits. Later, suspecting kwashiorkor, neighbours advised mother to seek care from local health facility.	Used to walk long distances to seek care, so as to reduce costs. Missed meals or reduced intake to help cover expenses for the child during treatment seeking. Siblings stopped schooling during the child’s admission. Post Could not sustain providing nourishing food as prescribed at discharge.	Received support from relatives, neighbours and friends in different forms: advice, loans or foodstuff. Neighbours convinced the child’s father to accept biomedical care and send funds for the same.	Took long to diagnose the problem despite several visits to local health practitioners and health facilities. Some levels of mistrust (local hws) regarding post treatment therapy. Couldn’t access care when needed during a health worker strike. Sometimes had to self-medicate due to regular drug stock-outs at local facility.	Had initially been referred to a different subcounty hospital. But chose to go to KCH as was unfamiliar with that facility and town in which it is located.	Ensure child fed on nutritious food: fruits, high protein content foods-eggs, milk though couldn’t sustain. Also, asked to observe and maintain hygiene around the child- limited water sources around her area.

We drew on both the narrative and coded data to identity forms of vulnerability of (re) admission, prolonged illness or death (intrapersonal, interpersonal, environmental and structural), and agency, observed at household/community levels and in health service interactions.

### Ethical considerations

CHAIN protocols were approved for science and ethics in all participating countries. Collaboration with REACH supported us to embed ethics research within the CHAIN cohort study. The REACH Principle Investigator (MK) is the CHAIN ethics advisor who provided pre-study and on-going ethics advice and guidance. In Kenya, the research was reviewed and approved by the Kenya Medical Research Institute (KEMRI) Scientific and Ethics Review Unit (KEMRI/SERU/3318/054). Approval was also sought and received from the Oxford Tropical Research Ethics Committee (OxTREC number 34–16).

Working with families longitudinally can be ethically challenging given the forms of socio-economic and biological vulnerabilities anticipated. Still, this approach was valuable in generating the type of data needed to develop appropriate interventions. Verbal consent was sought from participants during admission to provide initial information and ask permission to visit homesteads. Written informed consent was then sought from participants on the first household visit for all in-depth interviews, observations and recordings. Consent was checked in subsequent household visits. As a form of appreciation and to compensate for time disruptions during our home visits, each household was provided with a food package after each visit. This was based on past experience [31] and was in line with the institution’s benefits guidelines. Where a child was observed to require medical attention during a research interaction, caregivers were referred to local facilities or CHAIN clinicians. In debrief meetings, ethical dilemmas encountered by frontline staff were raised and discussed, and where considered appropriate, acted upon.

## Results

Following a description of the 20 selected children and their households, we provide an overview of the treatment-seeking patterns revealed through household members’ narratives. Given higher post-hospital discharge mortality risks among undernourished children and our finding that children diagnosed with SWK had more complex treatment-seeking pathways than MW and NW children, we then examine the influences on treatment-seeking patterns for the 10 SWK households in more depth. We discuss the household/community level and health system/service influences for the pre-admission and post-discharge periods in turn, and in the discussion, draw out the forms of vulnerability and agency observed across the entire treatment-seeking pathway.

### Description of households and broad socio-economic situation

Basic data on the 20 children and their households are shown in Table 2. Children’s ages ranged from 3 months to 24 months on admission. Ten children were classified as those diagnosed with SWK, 2 with MW and 8 with NW; 2 of the NW children were diagnosed with a chronic medical condition (sickle cell disease and epilepsy). Most households were extended,^1^ with 5 to 12 members, typically including grandparents, in-laws, cousins and grandchildren. On admission to hospital, most primary caregivers were biological mothers. They were aged 19 to 38 years, had one to nine children, and most had no primary schooling or less than 8 years of primary schooling. Four had attended high school or college.

**Table 2:** Description of Household data

PID	Mum Age & No. of Children	Child Age (Months)	Index child sick since birth?	Other illnesses/ diagnoses	Mum Marital status	Mum Education	HH structure & Size	Social disruptions before admission	Level/source of income	If slept hungry
**Group of Children with Moderate Wasting**										
**HH10**	25 (1)	3	Yes	Gastroenteritis	Married	Primary	Extended (7)	Y	Husband- Waiter Grandfather- Watchman	N
**HH16**	21 (1)	8	No	None	Single	Secondary	Extended (3)	N	Brother-casual	N
**Group of children with No Wasting**										
**HH5**	28 (1)	16	No	Gastroenteritis	Single	Secondary	Extended (12)	Y- child relocated to rural, change carer	Self-Runs cafe	Y
**HH6**	30 (3)	20	No	Fever of unknown origin	Married	Primary	Nuclear (6)	N	Husband- Banker Self-Business	N
**HH8**	19 (1)	11	No	Gastroenteritis	Married	Primary	Nuclear (3)	N- IDI said stopped working	Husband-construction	N
**HH9**	23 (1)	14	No	Sickle Cell Disease	Married	College	Extended (8)	Y- Mother sick	Husband-casual	N
**HH12**	23 (1)	23	Yes	Epilepsy	Single	Primary	Extended (11)	Y- Mother new job	Self-casual	N
**HH13**	38 (3)	6	No	LRTI	Single	Primary	Extended (7)	N	Self-fishmonger	Y
**HH14**	20 (3)	15	No	Meningitis	Married	Primary	Extended (11)	N	Grandfather-employed	N
**HH18**	29 (6)	18	No	Malaria	Married	None	Extended (9)	Y- Death of sibling	Husband- Watchman	Y
**Group of Children with Severe Wasting or Kwashiorkor**										
**HH1**	25 (1)	14	Yes	LRTI	Married	College	Nuclear (3)	Y- Mother stopped working	Husband-Casual	N
**HH2**	20 (2)	24	Yes	Gastroenteritis	Married	Primary	Extended (7)	Y- Birth of sibling	Husband & brother- watchmen	Y
**HH3**	20 (4)	21	No	Sepsis	Married	None	Extended (9)	N	Husband-Palmwine tapper	N
**HH4**	36 (9)	18	No	None	Single	None	Extended (10)	Y- Pregnancy & child relocated	Self-sell charcoal Grandmother- sells palmwine	Y
**HH7**	32 (3)	8	No	None	Single	Secondary	Extended (12)	N	Self-salonist	N
**HH11**	21 (1)	15	No	None	Separated	Primary	Extended (9)	Y-Mother & child relocated	Bro & inlaw-Masonry& casual	Y
**HH15**	19 (2)	21	No	None	Widow	Primary	Extended (11)	Y- Caregiver changed	Father & bro- Masonry	N
**HH17**	19 (3)	24	No	None	Married	Primary	Extended (10)	Y- Mother & child relocated	Husband-fisherman	N
**HH19**	21 (3)	15	Yes	Gastroenteritis	Separated	Primary	Extended (5)	Y- Parents separates & relocated	Self- Casual	Y
**HH20**	29 (4)	13	No	Cellulitis	Married	None	Extended (10)	Y- Mother relocated	Self-casual jobs	Y

Drawing on our detailed household summaries and narratives, we categorised households based on their overall socio-economic status, taking into consideration levels of income in the household, regularity of income, maternal education, maternal access to household income, and strength of social networks and support. Broadly, we noted lower socio-economic status among SWK compared to MW/NW children, with 6/10 and 3/10 being categorised as of very low socio-economic status respectively. Most mothers of children who had been diagnosed with SWK were unemployed, depending on their husbands or other male family members for economic support. Those engaged in income-generating activities were involved in low paying casual work including housekeeping jobs, selling charcoal or fetching water/firewood for neighbours, and–for men–harvesting and selling palm wine, masonry, or fishing. One of the women with a college education had been formally employed as a nursery teacher but had to leave her job due to the child’s illness, as will be discussed further below.

### An overview of treatment-seeking pathways

**Fig. 2:**
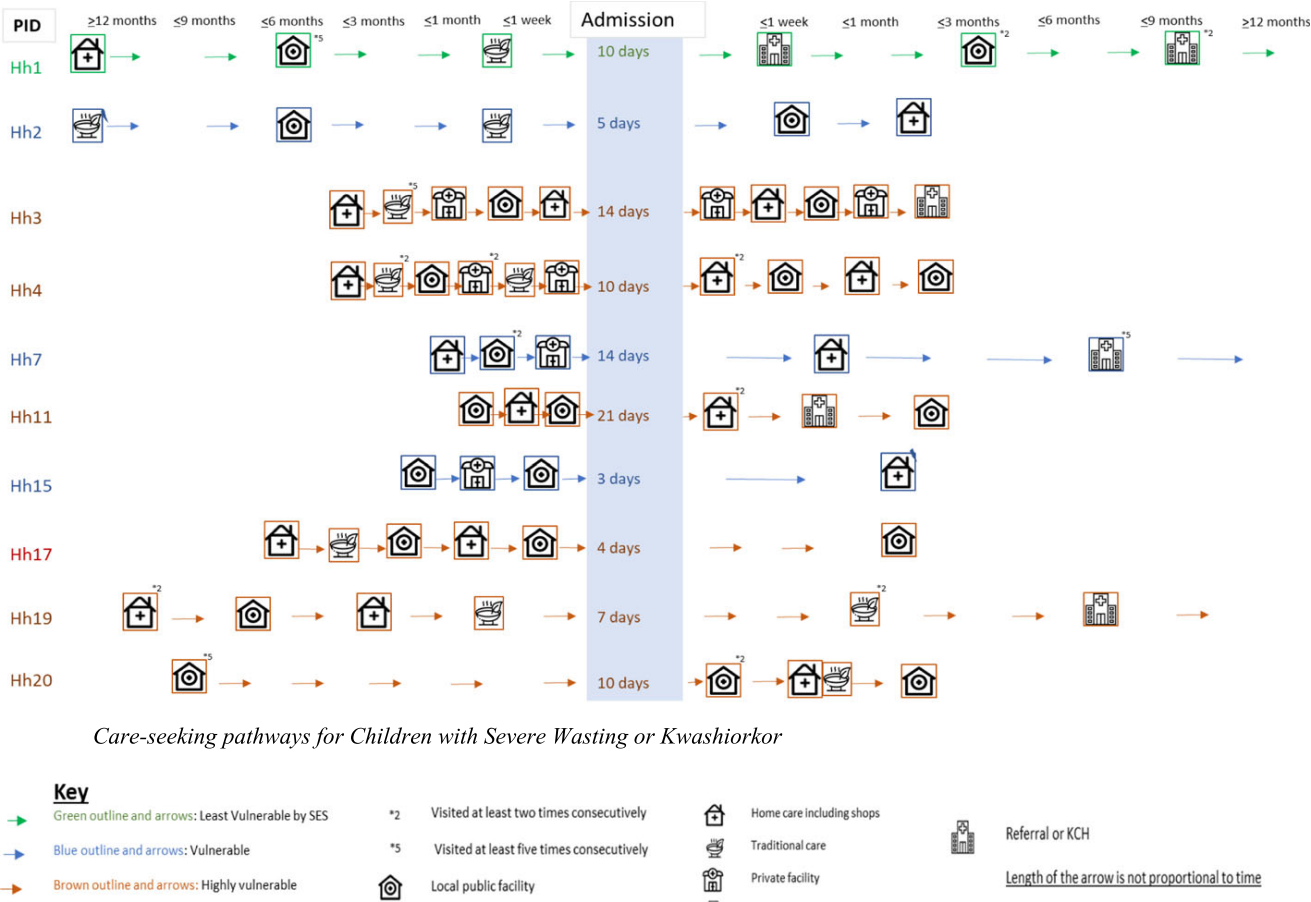
Care-seeking pathways for Children with Severe Wasting or Kwashiorkor

**Fig. 3:**
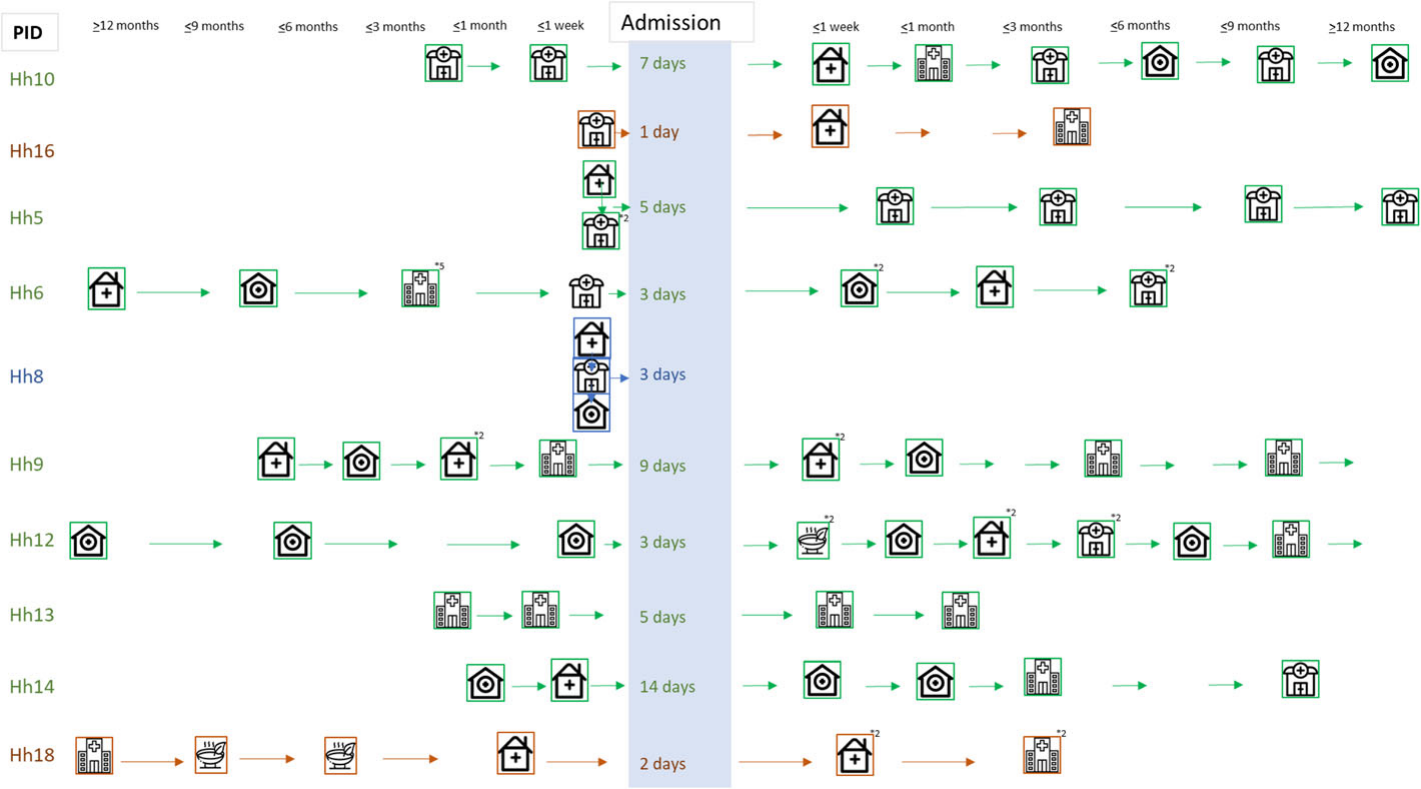
Care-seeking pathways for Children with Moderate Wasting OR No Wasting. Source of icons https://www.flaticon.com/

We developed visuals to summarise the length and nature of treatment-seeking for children’s illnesses pre- and post-hospital discharge (Figs. 2 and 3). These visuals are only indicative as caregivers were often unsure about when ‘this’ illness started and about which symptoms to consider a ‘new’ problem. They tended to describe one or several symptoms that developed and evolved over time. In some cases, a combination of symptoms and behaviours in the child triggered new concern about the child’s situation and (changes in) treatment-seeking action. Our documentation of recovery times is particularly fuzzy because our discussions focused on changes in illnesses/symptoms and treatment-seeking between visits rather than accurate timing.

The visuals illustrate that overall children who had been diagnosed with SWK broadly followed longer and more complex treatment-seeking pre-admission pathways than the other 10 children. The longer the illness had gone on for, the more treatment-seeking actions had been made, and the greater the likelihood of traditional or faith healers and private health facilities being visited. In the most complex pathway, we see over 6 formal health facility visits to try to treat a child before the hospital admission. Following hospital admission periods of between 3 and 21 days, most children with SWK were reported to have recovered within 3 months of discharge, but three were reported to still be unwell or to be ill again, by the end of our follow-up period. All caregivers reported visiting their local facilities for the regular child monitoring clinics after hospital discharge, and all attended the regular study follow-up clinics at the research centre. A further seven caregivers mentioned continuing to treat their children with home remedies or locally bought drugs. One of the ten caregivers discussed seeking traditional medical care for her child and another visited a private clinic.

While helpful for seeing important patterns in the care journey, these visuals cannot do justice to the richness, complexity and diversity of household’s situations and treatment-seeking stories; the combinations of stress, vulnerability, resilience and agency revealed. To bring to life some of these stories and provide a backdrop to the influences and experiences shared next, we include three different stories from families with children who were diagnosed with SWK (HHs 1, 3 and 20) in Additional file 1: Boxes 1, 2, and 3.

### Factors influencing treatment-seeking pathways and experiences for children admitted with malnutrition

Among children diagnosed with SWK, a range of factors influenced households’ decision-making and experiences across the treatment-seeking pathway. We share these processes and experiences for the pre-admission and post-admission periods in turn, highlighting household/community level influences and health service/system influences respectively. However, the pre- and post-discharge influences are inter-related, and many influences cross-cut entire pathways.

#### Pre-admission influences on treatment-seeking – household and community levels

##### Perceptions of illness causation and severity

Some symptoms such as fever, cough, running nose and diarrhoea were often considered by family members to be ‘normal’ for that stage of the child’s development at first. At this stage, symptoms were simply observed or treated using shop-bought drugs, with treatments most often mentioned for fever being paracetamol, and, for diarrhoea, oral rehydration solution. Caregivers associated diarrhoea with teething in particular:

*“He was teething. Now during teething normally, the child has diarrhoea and the diarrhoea stops once the tooth emerges. But with him, the diarrhoea never stopped even after the tooth has emerged … it would stop for two days and then start again ...[as] the teeth were emerging one at a time... [H] e was born in February and from June, July and August [this went on] … When we moved here [relocated to maternal home], he still had diarrhoea and then his health started to deteriorate.” Mother.HH19.*

This quote suggests that symptoms seen as associated with teething can continue for some time, as also observed in HH 20 (Additional file 1 Box 3). Across households, parents tended to take additional action where symptoms persisted or changed, or where the child’s condition or behaviour led to mounting concern. HH 3 (Additional file 1 Box 2) for example mentioned being triggered into action when her febrile child lost his appetite and started to have diarrhoea and vomiting:

*“Yes, so the fever continued for some days, but still he ate when I fed him. So, this continued but later on, he refused to eat, and that is when I realised that the illness was worsening … , he refused to eat and started [to have] diarrhoea...and vomiting. So, this continued, and I started thinking that my child is very ill now.” Mother.HH 3.*

In HH 3’s case, the first concern was that the vomiting was caused by a swollen uvula which the husband arranged to be removed by a traditional healer. In this home, as elsewhere, this and other actions were linked to ‘what is normally done’ for the symptoms observed:

*“The way we live here when a child’s body temperature rises, we just buy drugs from the shops. So, the child should get well after using the drugs, but if not, we go to a traditional healer for ‘mburuga’ [diviner].” Mother.HH3.*

While the use of shop-bought drugs as a first step was common across households, the next step varied. In HHs 1 and 20 (Additional file 1 Boxes 1 and 3), the next step was to bring the child to government biomedical facilities rather than healers. Nevertheless, healers were visited before admission in all three households, and by six out of the 10 households with a child diagnosed with SWK. Family members explained that among the Mijikenda community, some symptoms (wasting, dry or wrinkled skin, folded or crossed arms and legs), can be as associated with ‘*chirwa*’, said to occur if a parent is unfaithful during pregnancy [22]. When a child has these symptoms, families chose (or might be advised by others) to visit a traditional healer for diagnosis and help. Two of our 10 households mentioned having concerns about *chirwa* and visiting a healer at some point. As the college educated mum from HH 1 explained: “a*s a parent, you will just do whatever you think can to help the child*.”

Notable across our 10 households is that several caregivers (e.g. HH 3, Additional file 1 Box 2) seemed confused and unhappy with the diagnosis of malnutrition on admission which they attributed to ‘not having enough food’.

##### Levels of access to immediate cash

In this low-income context, lack of rapid access to money was raised as a key factor contributing to the child’s deteriorating condition and influencing when a treatment-seeking action was initiated, where and by whom (all households).

Most primary caretakers of the ten children were not income earners themselves, having to rely heavily on spouses or other household providers for money:

*“It is her husband [who provides] ... If we are ever short of funds or have none, she informs him that we have a particular problem here. Then he sends us some money which we withdraw [by mobile], and we can get food to eat. If he says that he does not have any, then we will have to sleep hungry because that is just life, there are ups and downs.” Grandmother.HH2.*

Even when mothers had the support of others, financial challenges were a major concern that had significant influence on treatment-seeking. Coping strategies included borrowing from relatives, friends or neighbours (five HHs), and asking for services on credit to refund later (three HHs). While these strategies often assisted families in seeking either biomedical or traditional care, subsequent repayment of loans was often difficult. Where the treatment taken was considered unsuccessful, families described regretting these actions.

*“I never saw anything good [about getting the treatment on credit]. In fact, it did not assist in any way. In fact, it worsened the situation …*. *thinking on the money he had charged and the diagnosis he made, I have done what he asked me, but the child’s condition remained the same. I felt very bad, and I wished it were possible for me to go back to him and demand for my money because he cheated me. Unfortunately, that is not possible.” Mother.HH3.*

Most family members described negative implications of treatment-seeking and associated costs for the household’s socio-economic stability in the short term, and four households described a longer term negative impact. For instance, where the caregiver was the main provider in the household, time off work to care for a child impacted on household income, in some cases necessitating the household to forego meals (three households), take older siblings out of school (two households), sell household items (two households), get basic goods on credit from local shops (three households) or delay paying rent (one household).

##### Social support–family and friends

Regular involvement of family and friends in discussions about ‘what was wrong’ with a child, and about when and where to seek care, was evident in all narratives. Levels of engagement were shown to increase when symptoms were unusual or became more alarming to primary carers, and when intended treatment-seeking actions were more distant from homes and more expensive. In some cases, the primary care-taker was persuaded by others that the child was unwell or needed a different form of help (e.g. HH 1, Additional file 1 Box 1); in others, the mother sought advice from others (see HHs 3 and 20; Additional file 1 Boxes 2 and 3).

Husbands and mothers-in-law played particularly important roles, especially where a husband had control over household resources and where a mother-in-law was either the primary decision-maker in the household or was perceived as experienced and able to provide good counsel:

*“There are factors to consider here. If you want to go to the hospital, everyone in this household should be informed. You cannot just leave without anyone’s knowledge that you left with a child who is unwell. You need to plan with everyone to get fare …*. *[With the mother in law we] even convinced the father to send money for fare.” Mother.HH3.*

Friends and neighbours stepped in to support with the care for the sick child or his/her siblings, to provide small gifts or loans of money and other essential goods (such as flour or salt) and advised on where and how to seek care. Others were most likely to step in when initial efforts by the family had not succeeded, or when the child’s mother did not appear to neighbours to be ‘doing enough’:

*“I had not thought of going to the hospital, but my neighbours were persuading me to go … [they explained] ‘at this stage you have to take the child to a certain place’. They even talked to the father until he thought of sending money for fare.” Mother.HH3.*

*“It’s the neighbours. They see, and they start talking … You know when people from this area see a child who is looking like that [losing weight] they say maybe you have done something …*. *I don’t know umechirwa [been unfaithful while pregnant] … that you have to take the child to a witch doctor especially because the child is too small.” Mother.HH1.*

Sometimes this social support was reported to be a burden and to cause delays in care-seeking or to create an additional financial burden for households. For instance, while the involvement of husbands and in-laws or grandparents was often appreciated and valued, there were some delays associated with the process, with many references made to ‘waiting to talk to my husband’, ‘waiting to be sent money from my husband’ and waiting for ‘his permission to pursue an action’.

It was notable that three of 10 women reported having experienced physical violence of some form from their child’s father. Mothers-in-law and neighbours also could be perceived as both helpful to treatment-seeking through intervening to demand a respond from the child’s father, or to introducing delays with unhelpful advice.

#### Pre-admission influences on treatment-seeking - health system/service level

##### Experiences with health centres and dispensaries

Proximity to and familiarity with health facilities played a significant role in determining where biomedical care was sought. For example, one child (HH 20) became unwell while visiting her grandmother in the city of Mombasa (approximately 60 kms from Kilifi) but returned to her rural home to seek care in more familiar facilities.

Government health centres and dispensaries are often cheaper than small private clinics and so were sometimes preferred by households living near them. Some families reported positive experiences at these facilities before admission and having only gone once or twice before being referred on to Kilifi County Hospital (KCH) for ‘proper’ diagnosis and treatment. However, others complained about the inability to get services on credit, and about the quality of care offered at public health facilities, especially health workers’ skills and attitudes:

*“They are arrogant, they ignore people even if somebody is serious like now my baby’s eye was turning yet somebody was not paying attention. Then when you reach there they touch the baby for fever and just write. [They] don’t want to do investigation to know exactly what’s wrong with the baby. If you say something, they’ll tell you that you talk too much. So, you find it difficult for them to help your child …*.” *Mother.HH1.*

Some families stopped visiting public facilities close to their homes as a result. For example, the mother in HH 1 (Additional file 1 Box 1) visited several public health facilities and was always concerned that the child was not carefully looked at, just given drugs. About one health centre she mentioned:

*“I met the doctor, and I told him/her to look at my child, and s/he started asking me [in front of everybody], “do you even take the time to feed this child?” I told her/him yes, “then comparing your child with other children, are they of the same size?” Ah, I walked out and came back home.” Mother.HH1.*

This mum was particularly upset by the suggestion that she was not trying to feed or help her child, explaining *“no parent would intentionally let her child suffer”.* Travelling to further facilities did not always yield positive results; in fact, some said it delayed recovery and added to cost burdens. Another delay was reported to be where children were referred to Kilifi County Hospital (by friends, family or health facilities), but where funds for travel and anticipated hospital fees were not available.

##### Reaching the hospital and being admitted

Three of the children classified in the SWK group had been referred to KCH from their local health facilities where health workers had felt the illness or symptoms were ‘beyond them’. The other seven families made their own decision to go, usually having had several unsuccessful visits to their local facilities. Some parents reported a smooth arrival and admission to the ward from the outpatient department and relief that their children were (finally) in ‘the right hands’. Nevertheless, many reported not being given clear instructions on where to go for admission itself. One mother described her experience:

*“I left [home] very early even before taking my breakfast, I thought I would be treated and come back, I didn’t know that I will be admitted. I spent the whole day in Kilifi up to four pm is when I was taken to see the doctor. I explained the problem, then the doctor never told me that I was admitted, he told me to wait for him, and he gave me the paper. So, I waited there up to 5 pm. [At that point] I asked another lady to help me in reading the paper, and she told me that the child was admitted, and the doctor has left. I saw another boy, and he was told to take me to Ward One.” Mother.HH1.*

Another referred mother (HH 20; Additional file 1 Box 3) reported being unfamiliar with the hospital and not knowing where to start; “*that’s why I kept on moving around here and there”.* By evening she gave up and decided to go home until a security guard on the exit gate looked at her papers and told her where to go. These data suggest that some children may fail to be admitted even if they have been referred from another facility to the hospital.

#### Post-discharge influences on treatment-seeking

##### The admission experience, including care, costs and advice

Many of the children classified in the SWK group were admitted for more than seven days. Parents described appreciating that over that period that their children showed signs of recovery, with most attributing that recovery to health workers being friendly, taking time to listen to their questions and worries, and paying close attention to their child’s condition and needs:

*“The doctors used to come check on them and inject drugs and give milk as required. If your child had any problem, you would tell the doctors because they did daily morning rounds to check on the progress of the child and see whether the doses needed to be changed/adjusted...” Mother.HH1.*

Children’s recovery was often reported to continue post discharge as a result of the good care in hospital and where they were able to follow the advice given during admission. Many carers appreciated advice on hygiene around the child, and on nutritious foods (meat, milk, eggs and fruits). The RUTF given and prescribed on discharge was mentioned less often; described more as a food supplement than as an essential requirement. This may be linked to a concern about how substantive therapeutic feeds are: four households mentioned that children should be given food in the hospital, not just the ‘milk’, or therapeutic feed. For example, the mother in HH 1 (Additional file 1 Box 1) complained that whenever she told the staff her child was not getting full with the milk, they would increase the amount a little, but did not add any solid food.

The socio-economic challenges many families faced before admission were in some cases compounded by the admission experience itself, with families being required to buy disposable nappies, utensils and supplementary food. Nappies cost about KShs. 20 [$.20] each and have been introduced by the hospital to minimise cross-infection and cleaning but are clearly a major cost burden for the poorest households. Further cost concerns during admission included ongoing needs of other children at home and anticipated charges on discharge. In the ward, parents supported one another practically (for example helping with laundry, use of phones to speak to family or receive money, or going to the shop), emotionally (for example encouraging those with ‘difficult feeders’ to keep trying) or with advice (for example on appropriate food to give children once discharged), and occasionally with cash. But financial costs were usually met by drawing on savings or borrowing from friends, relatives, and local savings groups, with longer-term implications. Many families also, therefore, did their best to minimise expenses not only for the sake of the admitted child but also others in the home:

*“I was telling the relatives because of bus fare, that instead of using the fare [using money to visit them while in admission] it’s better for them to buy food … So, I preferred they send me money [for diapers and bananas for the baby] because it was important for people at home to feed too. So, we all made some sacrifices.” Mother.HH7.*

Several adult family members at home had to forgo meals during the child’s admission, and one family mentioned that the index child’s siblings sometimes had to miss meals and school during the admission period.

Given this context, it was notable that only a few carers mentioned having been given advice on relatively cheap nutritional options such as beans and small local dried fish to grind with maize to make nutritious porridge. Also notable was some parents reporting being upset by hospital cleaners not showing respect to their belongings and ‘pushing them around’, and food servers occasionally shouting at or failing to serve some parents. One food server was reported to have accused mothers of only being in the ward to access free food when they should be at home earning an income. This was particularly upsetting for one mother who said she had cried in the ward because of her desperate need to fulfil many responsibilities at home rather than be “stuck in the ward”.

Although most parents were able to pay the hospital bill on discharge, this was usually after concerted efforts with relatives and friends using the strategies mentioned earlier in the paper. Two families reported having to stay in the hospital for several extra days post-discharge because they could not find the money to pay.

*“He [the child’s father] never had the money at that time. We had been discharged on a Tuesday, but we stayed there until Friday; that’s when we went home.” Mother.HH17.*

##### Returning to challenging socio-economic situations in homes

Families usually returned to challenging socio-economic situations in their homes and communities, in some cases compounded by the direct and indirect costs of their treatment-seeking journeys to date, and the admission itself. Although some families were able to follow advice on recommended food and hygiene for their children post-discharge, several were clearly very upset and frustrated that they could not afford to provide a better diet (for example HH 3, Additional file 1 Box 2), and at having to share whatever the recovering child needed with others in the home. As one mother explained:

*“What could I feel when I have failed to get what I was supposed to give my child? Yes, I will have to give some of his food to his siblings to eat. I was told the same thing in our local health facility [to ensure the child’s food is not shared with siblings but] I told them, honestly speaking like right now while I am here getting him checked up, I am not doing any work. So, where will I get that money to buy chicken at fifty shillings for him or fish, when the other siblings need to eat too? When I get like these fifty shillings, I can buy food at fifteen shillings for each of them so that they eat and then I will have to see how it will be in the evening.” Mother.HH19.*

Three mothers talked movingly about the harsh reality of having to choose between earning money to feed all of their children and having to care for the sick child. The mother in HH 1 (Additional file 1 Box 1) described a vicious cycle post-discharge. In essence, she could not return to her job because of the child’s health needs and was then unable to afford the transport costs for the occupational therapy recommended for her child. In this case, the main challenge for the mother with feeding and caring for this child was not so much the cost or access to food or even access to social support, but that he was a very poor feeder, something that our interview team also observed on visits to the home. Her frustration with being unable to feed her child adequately contributed to her feelings of helplessness, with her disappointment and irritation impacting back negatively on her persistence in feeding the child.

Primary caregivers in three households chose to move the child into another household to try to ensure that he/she received adequate care or nutrition, or to improve the child’s environment. In one case this appeared to be helpful, in the other two less so. A positive example was a child being sent to live with an aunt in Mombasa for 3 months to separate her from a younger sibling and enable her to access the recommended nutritious food. A less positive example was a child and his mother moving to her brother’s home where they were considered ‘visitors’ and refused food from the sister-in-law, even when she was aware they had no other source of food, which caused family arguments and rifts.

Whether children moved or stayed at home, it was notable that treatment-seeking actions for cough, fever, diarrhoea, vomiting or skin infections continued to be primarily based at home, using shop-bought drugs and herbs, for similar reasons to pre-admission and as outlined in the next section. One of the three women who sought treatment from a traditional or faith healer after discharge attributed the child’s slow recovery to his father’s transgressions. According to the mother, the child continued to experience episodes of diarrhoea and showed signs of delayed milestones. She visited two different healers to try to remedy the problem:

*“When I got back here [after hospital discharge] his condition had not yet improved so I wondered about what I should do, I took him to another traditional healer who performed some rituals to cleanse him. Mother.HH19.*

##### Continued concerns about the quality of care in other public facilities

For many families, concerns about the quality of care at local facilities persisted post-discharge, including concerns about the availability of staff and drugs, and staff attitudes. One participant lamented that she would rather buy drugs from a local shop than go to a facility that suffers from frequent drug stock-outs.

*When you go to the health facility, there are no drugs [pause] you are prescribed drugs and told to buy from a chemist, but you have no money to spend at the chemist. So, you come back home without medicines and when the conditions worsen you go to a duka to buy drugs. Mother.HH3.*

A specific issue reported by caregivers was conflicting information and advice being given by health workers at different levels of the health system. For instance, in HH 1, the mother mentioned she had been told by a health centre health worker that giving the child therapeutic feeds only, as done and advised during admission, was wrong:

*“When I went to the local health centre, the nutritionist at the place asked me, “do you mean that the child wasn’t given any solid food when he was admitted?” I told her that the child was only given milk. She further asked “You mean he wasn’t given potatoes? Potatoes with some meat?” Mother.HH1.*

This mother placed greater trust in the hospital advice and therefore avoided re-visiting the clinic, highlighting the importance of consistency and clarity in information across the health system. Many families who had been advised to replenish therapeutic feeds at the nearest local facility reported stock-outs when they went to collect supplies:

*“They [plumpy’nuts] got finished, and I was told to go back again. When I got there, they were out of stock, so I was given another date. But when I went again, they were still out of stock. That is the time when the doctors were on strike, so they told me that according to her weight, I should focus on providing her with nutritious food.” Mother.HH17.*

Access to facilities and supplies was particularly difficult for families during part of our fieldwork period because of public health sector strikes.

## Discussion

We gathered in-depth qualitative narratives from family members of hospitalised young children on their children’s treatment-seeking journeys. These narratives provide important insight into children’s vulnerabilities to poor outcomes (such as recurrent serious illness, (re) hospitalisation, disability and death), and family members’ agency and positive experiences. Drawing particularly on the narratives gathered from family members of children diagnosed with severe wasting or kwashiorkor, we learnt about the diversity of experience across households, and what were often complex care-seeking pathways, as well as the multiple layers of vulnerabilities faced by families as they navigated through those pathways. Here we discuss some of those vulnerabilities, and how they interplayed with parents’ and caregivers’ agency and the implications for future research and practice. In so doing, we follow others in noting that vulnerability encompasses ever-changing situations which interact with individuals’ ability to persist through challenging circumstances [13, 14, 16, 17].

Family interpretations and actions combined different sets of ‘recipe responses’ (doing what is normally done with the evolving (set of) problems), shaped by vulnerabilities and agency in constrained contexts [32]. Drawing on our narratives and the literature, we show in Table 3 the main situational or immediate vulnerabilities and indications of agency at household/community and health system levels.

**Table 3:** Situational Vulnerabilities and indicators of agency revealed through parent initiatives

	Vulnerabilities of children to (re) hospitalisation or death	Indications of agency in an effort to help the child recover
Within the household and in the wider community	**Intrapersonal** - Main carers’ and others’ poor physical and mental health and well-being - Anxieties about the health and well-being of the child **Interpersonal - Complex family situations and dynamics** - Family members living in households split geographically: access to main income earners difficult - Changes in living arrangements with negative implications for the child e.g. birth of a younger sibling leads to cessation of breast-feeding - Main carers often do not have decision-making power over household resources and treatment-seeking actions. Delays in seeking care can result from advice or demands of important decision-makers, including husbands and the parents (in-law), especially grandmothers - Some main carers face psychological or physical abuse from other household members **Environmental – socio-economic and cultural** - Lack of rapid access to funds and competing demands for those funds: Having to skip meals; cannot provide/sustain recommended foods; Low income restricts amount can borrow and get on credit - Finances required to travel to facilities, or to pay for facilities not available, or accessible to main carer: can delay treatment-seeking action, and those actions can further impact on income level or ability to earn - Perception of disease severity or cause leads to delays: Symptoms perceived as normal for prolonged periods, or to require treatment from healers	**Intrapersonal** Observed in the many treatment-seeking actions taken by mothers, and their determination to do the best they can in their circumstances: - Visiting many facilities - Shifting, repeating and mixing sources of care as seen necessary and appropriate - Accessing care on credit **Interpersonal - Accessing support from others in the home and community** - Seeking out and acting on advice on where to seek help for the child - Negotiating to secure funds or loans from husbands, others family members and neighbours - Working with for e.g. mothers-in-law and neighbours to convince the husband of the need for money or for a treatment-seeking action - Negotiating for delays in paying rent or pulling other children out of school to save money - Seeking and giving practical, emotional and advisory support from other parents **Environmental – socio-economic and cultural, and institutional** - Avoiding certain facilities as perceived to offer poor quality - Reorganising living arrangements such as moving child to live in another home - Rethinking foods giving, feeding arrangements and hygiene practices in the home - Seeking extra and cancelling work as needed or possible to help meet treatment-seeking needs - Demanding information and support from health providers, cleaners, security guards and others in health facilities
Interactions with health facilities and other similar institutions	**Intrapersonal** - Emotional and practical concerns about the child, quality of care, costs and needs of others **Interpersonal - Power relations between some staff and parents** - Being treated with disrespect can lead to fear to ask questions or share necessarily information with staff - Parents unable to demand more attention for their children, and lack of trust in care and advice given **Environmental – socio-economic and cultural, and institutional** - Lack of familiarity with, cost or distance from desired health care services leads to delay in access - Perceived poor quality of care – either technical or inter-personal - Cost burdens adding to family concerns, and – where incurred – to low availability of funds in households (e.g. transport costs, consultation and treatment costs, nappies and admission costs in hospitals**)** - Referral and continuity of care - recommended therapeutic feeds not available in facilities and being given conflicting advice; little mention of health care workers or community-based support	

There was clearly a wide range of vulnerabilities affecting the children, their families and the communities in which they live. Children’s biomedical conditions, and vulnerability to prolonged illness and (re) hospitalisation interacted with their families’ - and especially their mothers’ - intrapersonal (biological or psychological), inter-personal (roles, relationships and interactions), and environmental (socio-economic and cultural, and institutional) vulnerabilities.

In terms of intrapersonal vulnerabilities, we heard from many mothers about the emotional impact of their child’s illness, including worries about their child’s condition, confusion with symptoms, anger with perceived poor quality of care, and frustration with their inability to access therapeutic feeds or afford recommended nutritious foods. Receiving conflicting information from different public health providers about the adequacy and appropriateness of therapeutic feeds was particularly concerning for mothers, as was being made to feel responsible or to blame for their child’s condition by health providers or family members.

These emotional vulnerabilities influenced mothers’ decision-making and action pre- and post-hospital discharge in both subtle and overt ways and influenced whether the information given by health providers was valued and followed. Many mothers, for example, reported being angry with how they are talked to or their children are handled in health facilities and that they therefore avoid those facilities, distrust or ignore the advice given, or leave as soon as possible. These findings resonate with those from other low resourced contexts: concerns about poor inter-personal handling and health workers’ negative attitudes are widespread in the literature [10, 11, 33]. The critical importance in service delivery of recognising and responding to the communication and emotional needs of patients and their family members is therefore highlighted; dimensions of good quality of care are often under-supported in health worker training [33].

Environmental and inter-personal vulnerabilities fed into and sometimes exacerbated the emotional vulnerabilities of family members in homes, communities, and facilities. For example, socio-culturally, some delays in accessing biomedical care pre- and post-admission were influenced by symptoms being considered normal by family members for prolonged periods, or to require treatment from healer(s), sometimes over several weeks. Notably, ‘not having enough food’ was rarely described as the primary cause of a child’s problems. Others have also noted the importance of cultural perspectives and knowledge in understanding treatment-seeking patterns, and of identifying locally appropriate public engagement and education to build awareness of danger signs [22, 34].

Further vulnerabilities faced in our setting were socio-economic, with all 10 families of children diagnosed with SWK experiencing significant livelihoods challenges. These included low, irregular sources of income, competing demands on those resources, complex family situations and gendered family and community relations. Female carers’ control over assets, autonomy in decision making, and freedom from domestic violence have been found elsewhere to have an influence on childhood nutritional outcomes, including through influencing treatment-seeking [35]. We observed a vicious downward spiral between delays in access to preferred facilities and financial vulnerability over time: mothers often had to wait for funds or permission to seek services (usually from husbands or elders). There were often challenges in finding funds to cover both transport and treatment costs; and seeking care led to further costs, which then fed back into livelihoods challenges. These findings echo those from other under-resourced settings like Ghana and Tanzania that concerns about treatment-seeking costs can delay action, and when incurred, can be catastrophic for household survival [12, 36, 37]. They suggest the importance of identifying interventions that minimise both direct and indirect costs of care for potentially vulnerable patients, including malnourished children. Even ensuring that simple costs such as for nappies during admission are eliminated has the potential to have a positive impact on the most vulnerable families.

These multiple situational or more immediate, intrapersonal, interpersonal and environmental forms of vulnerability were shaped by broader structural drivers of children’s situations. In the Kenyan coastal context, structural vulnerabilities encompass scarce income-earning opportunities, impoverished formal education, seasonal drought and food shortages, highly resource-constrained public-sector health services, and strong gender inequities [21, 23]. These influences on parents or patients’ treatment-seeking are rarely identified in clinical encounters globally despite being known to be critical to health outcomes. Bourgois et al. [38] suggest that the development of a mixed methodology screening tool could help clinicians quickly gauge aspects of a patient’s structural vulnerability and whether he/she is likely to benefit from locally available services. In LMICs like Kenya, such an initiative could include making referrals to specific health and social services and supporting linkage to local non-governmental and community organisations.

Not all vulnerabilities highlighted in Table 3 were faced by all households, and it was notable that even within these serious, complex vulnerabilities we saw mothers and others exercising choice and making important decisions in their efforts to help the child. Although many mothers’ opportunities to exercise agency were heavily constrained by their social and financial circumstances, and by the broader context, they exhibited direct and more hidden strategies to exercise some choice and act. They demonstrated emotional resilience such as hope and trust in the face of significant challenges, reorganised their families’ living and feeding arrangements and enacted a vast array of treatment-seeking actions in their efforts to get their child seen and treated. In so doing they often had to mobilise advisory, financial, emotional or practical support from others.

The importance of recognising agency as relational (based on and shaped through interactions with others) and as constrained by prevailing social, economic and cultural situations is increasingly recognised in the literature [39]. Campbell and Mannell also note the importance of recognising less overt, discrete and identifiable actions in understanding agency, particularly in constrained gender unequal contexts. In our study, we saw mothers avoiding some facilities and indirectly persuading their husbands to assist through the intervention of others (such as neighbours and mothers-in-law). Campbell and Mannell’s work serves as an important reminder that in developing interventions to improve child outcomes, it is essential that existing initiatives and hidden efforts are recognised, that responsibility for action is not inappropriately placed on individuals, that important relationships are protected, and that collective, institutional and state responses are emphasised [39].

As has been found across a range of LMIC contexts [10, 22, 23], the ‘significant others’ most often involved in supporting mothers were the children’s fathers and female grandparents, and other female relatives and neighbours. Mothers’ agency and the advice and support they received from significant others were often positive. However, our findings confirm the importance of moving beyond the agency–vulnerability binary in understanding care-seeking experiences among families: expressions of agency can arise despite serious vulnerabilities, and vice versa and individuals can experience both simultaneously [16]. In our study, some actions by mothers appeared to be both an act of constrained agency and vulnerability, with the potential for both positive and unintended consequences. For example, deliberate household reorganisations post-hospital discharge (where children were sent to live with others) were acts of both constrained agency and vulnerability. While some had a positive impact on the child’s health and well-being, others appeared to be detrimental. Similarly, those offering social support could have both positive and less positive influences. While the child’s father could be invaluable in supporting with advice and funds, in other cases, he was absent, contributed to delays in care or was even violent. And while neighbours’ interventions could be invaluable, in some cases, mothers also reported feeling ‘looked down upon’ or ‘pitied’ because of the child’s condition and the perceived cause, or stigmatised. These findings emphasise the importance of taking significant others’ roles and family and community negotiations into account in planning health system interventions [10, 22, 23]. They also highlight the difficulty in developing simple quantitative indicators of vulnerability and agency.

## Conclusion

Family members’ treatment-seeking stories reveal that children’s carers, usually mothers, navigate diverse challenges related to a range of intersecting vulnerabilities at individual, household and facility levels in their efforts to support their children’s treatment and recovery. Their agency, while demonstrated in complex treatment-seeking pathways, and in the changes even the most vulnerable families introduced into their daily lives, was often significantly constrained by their situation and by broader structural drivers.

The findings support the importance of ensuring effective communication and context-appropriate advice from facilities and strengthening continuity of care in LMICs, including relational, informational and managerial continuity, particularly for chronic conditions [40]. All of the initiatives and activities by families, and especially by mothers, in the context of multiple and interacting vulnerabilities, indicate lost opportunities by the health system to offer earlier and broader support to children, mothers and significant others.

In addition to, and potentially as part of building continuity of care, health providers and systems must develop ways to engage with the political and economic forces outside the clinical encounter that have a crucial influence on outcomes; that is, on patients and their family members’ structural vulnerability. Assessment tools and appropriate protocols such as those suggested by Bourgois et al. [38] could be tailored to particular clinical institutions and community resource bases and must take into account gendered roles, relations and constraints. Such initiatives, if carefully co-developed with relevant local stakeholders, have the potential to serve as an awareness-raising and action tool to identify and respond to structural vulnerabilities encountered.

## Supplementary information

**Additional file 1.**

## Data Availability

The datasets generated and analysed during the study are not publicly available due to institutional rules and regulations but may be available from the corresponding author on reasonable request.

## Abbreviations

CHAIN: Childhood Acute Illness and Nutrition Network
LMICs: Low and middle-income countries
SWK: Severe Wasting or kwashiorkor
MW: Moderate wasting
NW: No wasting
REACH: Resilience, Empowerment and Advocacy in Women’s and Children’s Health Research
KEMRI: Kenya Medical Research Institute
SERU: Scientific and ethics review unit
OxTREC: Oxford Tropical Research Ethics Committee
HH: Household
KCH: Kilifi County Hospital

## Footnotes

1 In this paper and throughout our study, we adopted the definition of an extended family as set of individuals living together either related by blood or marriage including father, mother, their children, their grandparents, cousins and other relatives.
