# Supplementary material for: Vulnerability and agency across treatment-seeking journeys for acutely ill children: how family members navigate complex healthcare before, during and after hospitalisation in a rural Kenyan setting
Source: Int J Equity Health. 2020 Aug 10;19:136. doi: 10.1186/s12939-020-01252-x (PMC7418306; doi:10.1186/s12939-020-01252-x)
Supplement: Supplementary file 1 — Additional file 1. [file 12939_2020_1252_MOESM1_ESM.docx]

**Appendices**

**BOX 1** HH 1

**Baraka: A child aged 13 months on admission (HH 001) – least vulnerable socio-economically; child remains ‘a poor feeder’**

*The household set up and the child’s situation since birth*

This child lives with his 25-year-old mother, and his father, in an urban area which is 1 hours’ bus ride from the hospital. They have lived in the area for 2 years and have no relatives in town. The child’s mother is a college-educated nursery school teacher, and the father – who is supportive and engaged in the home - works in town. The child has been sickly and poor feeder from birth – with many coughs, colds, and fevers. Because the child has always been a poor feeder, the mum was worried about leaving the child with neighbours – instead carrying him to work every day, lying him down when he was asleep.

*Treatment-seeking actions and costs leading to admission*

The mum had sought treatment from a series of places over time, including the local shop, and then two different public health care facilities over several weeks. In the facilities, the child was rarely carefully looked at, just given drugs. In one facility, the mum was told off in front of everybody in the waiting bay about the small size of the baby compared to the rest. She was so upset that she left the facility without care, explaining *‘no parent would intentionally let her child suffer’.*

Following these efforts, and because the child was not getting better, neighbours said he might have ‘chirwa’ (a local illness with similar symptoms to malnutrition believed to be caused by a transgression between husband and wife). So, they went to a healer with the hope that this would facilitate the recovery of her child. After two weeks of the healer’s treatment which cost 1000 Kenya shillings (about $10), there was not much sustained improvement. Going back to a government facility again, she was then told the child was very underweight and referred to the admission hospital where he was admitted for 10 days. To pay for the transport and other hospital costs, her husband negotiated with the landlord to use the rent money.

*The admission and post-discharge experience*

Although they were generally very positive about their handling at the admission hospital, they had one complaint/recommendation: to give children food (not just milk). The mum said whenever she told the staff her child was not getting satisfied, they would increase the amount of therapeutic milk a bit, but not add any other food; he was only given the ready-to-use therapeutic feeds at discharge. She feels that this further reduced the child’s appetite post-discharge. Moreover, when she went to a local facility to get a supplements’ refill, she was asked why the child had not been appropriately fed on meat and mashed potatoes during admission.

Since discharge, the mum has not been able to return to work because of the child’s health, and she has discontinued the occupational therapy that was being given in the admission hospital because of transport costs to get there. Although the child is gaining some weight, it’s very slow and inconsistent. He remains a very poor feeder, and the mum reports it can affect her own mood, which in turn can affect her efforts to feed the child.

**BOX 2** HH 3

**Hamisi: A child aged 1 year 9 months on admission (HH 003) – highly vulnerable socio-economically; child has periodic health problems and not thriving**

*The household set up and the child’s situation since birth*

This child lives in an extended rural home with four siblings (three older, one younger), his 20-year-old mother, and the child’s paternal uncle and grandmother. His mum has no formal education and is unemployed. She relies on farming and her husband’s income, with some help from the grandmother. The father lives and works in another rural location on rented land; visiting home every 2 months or so depending on how his palm wine tapping business has fared. Our team noted that the household was very low income and often hungry, witnessing for example siblings scrambling over small amounts of food, that the whole family suffered from skin infections, and the mum not having ability to treat her own health problems. The child was of good health and feeding well until he was about 15 months when he started getting high fevers and coughs. The mother noticed he then started losing his appetite, vomiting and experiencing diarrhoea and later started to swell and develop skin sores.

*Treatment-seeking actions and costs leading to admission*

When the child started losing his appetite, vomiting and experiencing diarrhoea, she and the grandmother felt the child’s ‘kilimi’- uvula – was causing the vomiting. She took him to the father’s place of work and he made arrangements for it to be cut out by a healer. However, the symptoms persisted for a further month, so she took the child to a series of healers (3) who diagnosed possession by evil spirits and recommended treatment. However, he soon started to swell and so the mum consulted a retired health worker who told her the child’s blood was low. She was prescribed medication which did not work despite reassurances that it would. On visiting another retiree in a neighbouring village, the child was diagnosed with kwashiorkor and given an injection but started to develop wounds. On her way back to the practitioner for more help (and to pay an outstanding bill), the mother met a neighbour who advised her to consult another neighbour whose child had suffered similar symptoms. That neighbour advised her to go to a government dispensary and convinced her own husband to buy a cock from the mother so that the mother had the money to get to the facility. When the mother went to that dispensary, she was given some medication and a return appointment and on that second appointment, the child was referred to a government hospital in a nearby town. On consultation with the husband and following advice from neighbours, she decided instead to go to Kilifi hospital which is closer and more familiar. The father had wanted to continue seeking treatment from traditional healers all along, but the mother wanted to go to the hospital because she had been panicked by neighbours. They advised her that if she didn’t take the child to Kilifi hospital, she risked him dying. In the end, the mother and grandmother persuaded the husband about the need to go to the hospital. She’d already spent over KShs. 2300 [$ 23] on treatment and unpaid balance at one of the private practitioners.

*The admission and post-discharge experience*

The child was admitted for 2 weeks, treated for scabies and advised the child had kwashiorkor. The mum was happy with the care and advice given, and with the plumpy’nut prescribed, but unhappy and confused to hear about the kwashiorkor diagnosis, having cared for this child as for his siblings who had never suffered from it. Post-discharge, the mum was trying to follow the advice she had been given at the hospital to buy and feed the child baby milk, baby porridge, butter and eggs, and had started storing porridge in a thermo-flask to allow her child to eat regularly. However, this new diet could only be sustained for two weeks because of finances. The family still suffered periods of hunger, and the child experienced fever, several fitting episodes, a leg infection, and malaria since discharge (with our team intervening to support access to care and referral on several occasions).

**BOX 3** HH 20

**Khadija: A child aged 14 months on admission (HH 020) – highly vulnerable socio-economically, child had recovered by the end of the study**

*The household set up and the child’s situation since birth*

This child lives with one older sibling and her mum in their rural home, with the father’s relatives, including his mother, who is a traditional healer. Two other siblings (aged 8 and 4) live in Mombasa with their maternal grandmother. The child’s mother is married, with no education. She does casual work for a living, including cultivating others’ land, fetching water, and pounding maize. She used to burn and sell charcoal but gave up because her husband would take all her money. The mum has been living in the home for about 10 years (since she got married), living in the child’s grandmother’s compound (second wife of the child’s paternal grandfather). The father of the child is the primary income earner, working as a motorbike driver and helping take palm wine to his father’s bar. However, he faces challenges, having attempted suicide during our study period. He did this after an argument with the child’s mother because he drank the KShs. 600 [$ 6] his father had given him for his daughter’s school fees. The child was of good health since birth until at around four months when she fell ill with flu/cold and fever. Sometimes she would have very high temperatures and eventually got emaciated and diarrhoea.

*Treatment-seeking actions and costs leading to admission*

When the child was first ill, she was taken to a local health centre on several different occasions, where she was treated for malaria and recovered. The third time the child was taken to a local health facility, the tests were negative, and the child didn’t improve with the drugs prescribed. The mum returned to the facility even before the drugs were finished and was admitted overnight and given 2 injections. Although she recovered, she soon got sick again and was very emaciated (‘could be lifted with one hand’). The mum thought this was because of teething. The child was taken to the health centre many more times in that month with her drugs being changed several times. After the last of the visits, the condition did not improve, and before the end of that week, the mother took the child back to the health centre thinking its flu. During this time, the child had blisters on his feet, skin depigmentation, dermatitis and oozing sores all over her body. The health workers told the mother they could not treat the condition as they didn’t understand it and that she should take the child to KCH. She felt discouraged by this. Her friends and neighbours would also ask what the child was suffering from, and in the end, it was heart-breaking for the mother. It looked like it was a strange condition to them (friends and neighbours). The child was referred to KCH. The father initially did not agree for the mum to take the child to hospital, but the grand mum intervened, and so he eventually agreed. The mum stayed at home for a further week before admission because of these disagreements and because she could not afford the fare to the hospital. No one in the family or even from the neighbourhood touched the child because of the oozing wounds the child had all over her body.

*The admission and post-discharge experience*

She did not think that the child would be admitted. She arrived at KCH in the morning around 10/11 am. Since this was her first time in KCH, she did not know where to go, so stayed at OPD till almost 4 pm. She had given up and was leaving the hospital premises when a security guard at the gate helped show her a doctor who admitted her in the HDU. Once admitted, she appreciated all the treatment and support she was given. The doctors supported with treatment and advice (on child feeding) and even helped her by buying her a basin, cup, plate, spoon, some clothes for the child and two lesos. Her mother and the child’s grandfather assisted with some other funds to cover the bills and other day to day needs. Regarding following advice given during discharge, she was able to provide beans, rice, meat, milk and vegetables to the child for a short time, but then stopped due to lack of money. So, she switched to giving the child game meat and bought bananas for the child. The child appears to have recovered – the mum attributes this to the hospital treatment and advice.
